# Long-Term Remission After Matched Sibling Donor Hematopoietic Cell Transplantation in a Patient With Primary Cutaneous CD8+ Aggressive Epidermotropic Cytotoxic T-Cell Lymphoma

**DOI:** 10.7759/cureus.15132

**Published:** 2021-05-20

**Authors:** Quinto Gesiotto, Yumeng Zhang, Ayesha Malik, Lucia Seminario-Vidal, Ernesto Ayala, Ling Zhang, Lubomir Sokol

**Affiliations:** 1 Internal Medicine, University of South Florida Morsani College of Medicine, Tampa, USA; 2 Hematology and Oncology, Moffitt Cancer Center, Tampa, USA; 3 Endocrinology, Mayo Clinic, Jacksonville, USA; 4 Cutaneous Oncology Program, Moffitt Cancer Center, Tampa, USA; 5 Bone Marrow Transplant Program, Mayo Clinic, Jacksonville, USA; 6 Hematopathology, Moffitt Cancer Center, Tampa, USA

**Keywords:** primary cutaneous cd8+ aggressive epidermotropic cytotoxic t-cell lymphoma, cutaneous t cell lymphoma (cd8+ pcaectl), hematopoietic cell transplantation, bone marrow transplant, lymphoma

## Abstract

Primary cutaneous CD8+ aggressive epidermotropic cytotoxic T-cell lymphoma (CD8+ PCAECTL) is an extremely rare neoplasm with a poor prognosis. Chemotherapy typically does not result in a sustained response, and hematopoietic stem cell transplant (HSCT) is the only therapy that has been shown to produce a durable response of any kind. Here, we report a case of a 25-year-old previously healthy male who presented with a painful ulcerative lesion on the bottom of his right great toe and local lymphadenopathy. The biopsy of the lesion was consistent with CD8+ PCAECTL. He received immediate chemotherapy followed by matched related donor HSCT (MRD-HSCT) and remained in complete remission (CR) for eight years post-transplant, longer than any CR reported in the literature. In conclusion, our report provides clinical evidence that early transplant consult and donor search is one of the key factors in the management of CD8+ PCAECTL.

## Introduction

Primary cutaneous CD8+ aggressive epidermotropic cytotoxic T-cell lymphoma (CD8+ PCAECTL) is an extremely rare neoplasm, with aggressive clinical behavior. It accounts for <1% of all cutaneous lymphoma and remains a provisional entity in the 2018 WHO-EORTC classification [1]. It is characterized by widely distributed papules, nodules, plaques, and tumors, frequently with ulceration, hemorrhage, and necrosis [2,3]. Rapid progression with extracutaneous dissemination is the natural course of the disease, with systemic spread to unusual sites including the lung, testes, adrenal glands, and central nervous system (CNS) [1,4-6].

## Case presentation

A 25-year-old previously healthy man initially presented to his primary care physician with a tender subcentimeter red bump on the bottom of his right great toe. Six days later, his lesion became ulcerated and started bleeding. Despite proper wound care with silver nitrate and compression dressings, the lesion became more painful and expanded in size over the next three weeks although he did not have systemic complaints at that time. He saw a community dermatologist who did a biopsy of his right great toe revealing an atypical lymphoid cell infiltrate consistent with “T-cell lymphoma” without further subclassification. He was referred to our institution for a consultation two months after his initial presentation.

**Figure 1:**
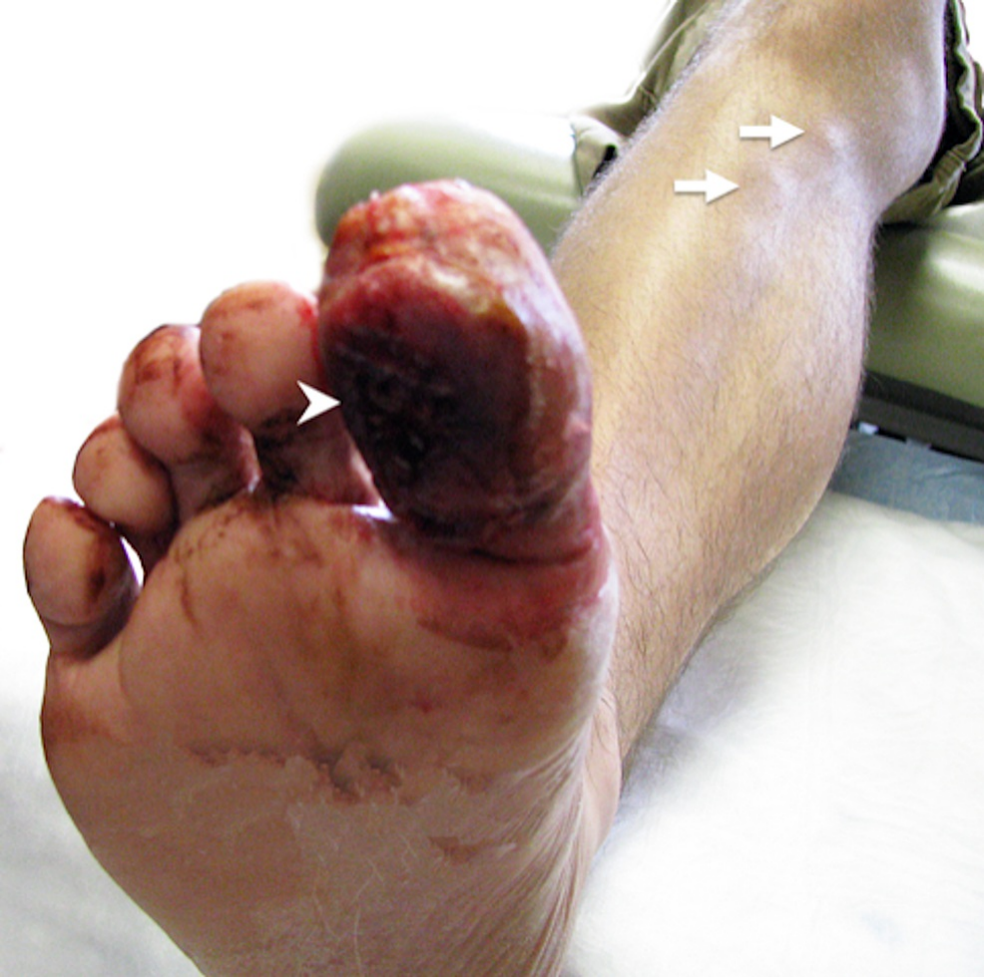
CD8+ primary cutaneous aggressive epidermotropic T cell lymphoma Vegetative ulcerated tumor on the plantar surface of the right great toe measuring approximately 4 cm (indicated by arrowhead). Three subcutaneous nodules along the ascending lymphatic pathway, a 1-cm diameter lesion on the dorsum of the foot (not seen in the figure), and two 1-cm lesions just below the knee (as indicated by arrows).

**Figure 2:**
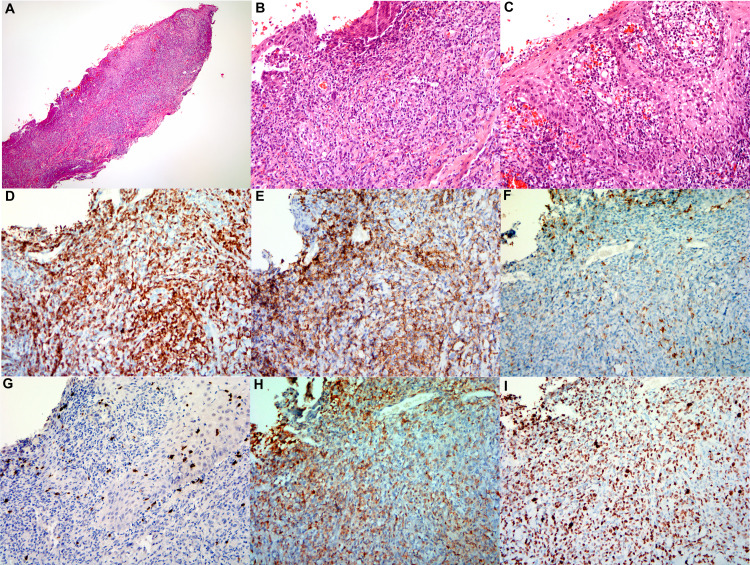
Biopsy of CD8+ primary cutaneous aggressive epidermotropic T cell lymphoma (A) Low power view of skin shave biopsy from the patient diagnosed with “primary cutaneous CD8 positive aggressive epidermotrophic cytotoxic T cell lymphoma. There is extensive atypical lymphoid infiltrate with epidemontrophism associated with “Pautrier microabscessess,” significant squamous cell surface showed erosion, ulceration with detachment or loss of epidermis (H&E, ×40). (B) Ulcerated skin surface with dense sub-epidermal cellular infiltrate composed of medium-sized lymphoid cells with dispersing chromatin (H&E, ×200). (C) Typical “pautrier microabscessess” with a collection of atypical lymphoid cells in the epidermis (H&E, ×200). (D)-(H) A panel of immunohistochemical stains was performed on the skin biopsy which confirmed that the neoplastic T-cells to be positive CD3 (C, ×200), CD8 (E, ×200), and TCR beta F1 (H, ×200) and negative for CD4 (F, ×200) and TCR delta (G, ×200). The estimated proliferation rate by Ki67 of 80%.

Physical examination showed an ulcerative tumor with associated hemorrhagic changes encompassing almost the entire right great toe measuring approximately 4.0 × 4.0 cm^2^ (Figure 1). Three additional subcutaneous nodules were observed along the ascending lymphatic pathway, a 1.0-cm diameter lesion on the dorsum of the foot, and two 1.0-cm lesions just below the knee (Figure 1). In addition, there was a palpable lymph node in the right inguinal area (1.0 cm). Laboratory studies with complete blood count (CBC) and complete metabolic panel (CMP) were within normal limits. Lactate dehydrogenase (LDH) level was elevated at 722 U/L (normal range: 313-618 U/L). EBV and HIV testing were negative. The staging PET/CT noted marked fluorodeoxyglucose (FDG) uptake in the right great toe, medial dorsal aspect of right foot, the anterior proximal tibial shaft, as well as right groin lymphadenopathy with a maximum SUV of 8.9 ranging from 1.7 to 8.9, consistent with physical examination findings. The toe biopsy, reviewed at our institution, showed intensive infiltration in the epidermal and dermal area by small to medium atypical lymphoid cells associated with extensive epidermotropism, significant squamous cell surface erosion, ulceration, and microabscess on the epidermic layer. Atypical lymphoid infiltrate extending into the deep muscular layer was associated with focal congestion and vascular proliferation (Figure 2). The immunohistochemical stains showed CD3+CD8+ atypical T-lymphocytes with partial coexpression of CD30, and lack of expression of CD5 and CD7. Ki-67 proliferation index was 80%. Blood flow cytometry was normal. These findings supported the diagnosis of CD8+ PCAECTL. Staging bone marrow biopsy showed no evidence of lymphoma infiltration and normal cytogenetics. Cerebrospinal fluid analysis was negative for malignancy.

Due to an aggressive clinical course complicated with arterial bleeding secondary to the vascular invasion of the great toe with tumor, he was started on one course of CHOP (cyclophosphamide, doxorubicin, vincristine, and prednisone) immediately, followed by involved site radiation therapy to the right great toe (3600 cGy in 20 fractions). In one month, he achieved a significant reduction in tumor size, and subsequently, he started on HyperCVAD (hyperfractionated cyclophosphamide, vincristine, doxorubicin, and dexamethasone alternating with high-dose methotrexate and cytarabine).

He completed six total cycles (three arms A/three arms B) of chemotherapy and achieved complete response as evidenced by clinical resolution of all previously observed skin lesions and PET/CT showing no areas of hypermetabolic uptake. Six months after initial presentation, he underwent matched related donor hematopoietic cell transplantation (MRD-HSCT). The conditioning regimen consisted of cyclophosphamide on day 6 and day 5 (200 mg/kg total dose) followed by fractionated total body irradiation (TBI; 1320 cGy in eight fractions, 2×/day) on day 4 to 1. Graft versus Host disease (GvHD) prophylaxis consisted of cyclosporine and methotrexate. Chimerism studies performed on the whole bone marrow cell compartment on day 28 demonstrated 100% donor chimerism, which was confirmed with an analysis of CD3+ and CD33+ cells in peripheral blood. A long-run clinical follow-up showed that he remained in complete remission (CR) eight years post-transplant.

## Discussion

CD8+ PCAECTL has a poor prognosis with a five-year overall survival of 32% in the largest multicenter retrospective study [1]. In that cohort of patients, Guitart et al. reported multiple treatment modalities used in 30 patients diagnosed with CD8+ PCAECTL, including chemotherapy, electron beam radiation therapy, and HSCT. The most commonly used chemotherapy agents included etoposide, gemcitabine, romidepsin, and liposomal doxorubicin. Despite the heterogeneity in management, no therapy showed significant survival benefits. Only 40% of patients who received chemotherapy remained alive by the end of the study. Overall prognosis with chemotherapy alone is dismal [1,3,5].

To the best of our knowledge, our patient had the longest CR of 96 months (as of April 2021) reported in the literature. The second-longest CR reported in the literature was 58 months on brentuximab monotherapy [7]. We believe four main reasons contributed to the success in this case. The first was the aggressive induction chemotherapy with HyperCVAD resulting in CR prior to MRD-HST. The second was the early transplant consult and prompt transplantation once the patient achieved CR. Third, the tumor was non-bulky and limited entirely to the lower extremity and regional lymph nodes. Fourth, the patient was relatively young at the time of diagnosis and did not have other significant comorbidities. While data about the optimal induction regimen are limited in CD8+ PCAECTL [8,9], the aggressive nature of the disease warrants higher intensity chemotherapy regimens to achieve remission as early as possible. However, larger studies are needed to confirm this observation.

Since chemotherapy alone usually is unable to result in a sustained remission, early bone marrow transplant consultation, and donor search are necessary for the improvement of outcomes in patients with PCAECTL. In the review by Guitart et al., HSCT was the only therapy that produced sustained response of any kind, as five out of six patients undergoing allogeneic hematopoietic cell transplantation (allo-HCT) achieved partial or CR with survival ranging from 16 to 168 months [1]. Disease status prior to transplant was not discussed in this study. A case series and systematic literature review by Cyrenne et al. emphasized the role of stem cell transplantation in CD8+ PCAECTL and showed superior outcomes in the eight patients who received alloHCT (CR 75%) compared to autologous transplant candidates at a median follow of 40 months (range: 6-171 months) [10]. In a more recent review by Ichikawa et al. [11], two out of three patients who received matched unrelated donor HCT (MUD-HCT) achieved CR [12]. Notably, none of these three patients were in remission before transplantation. There is a case report of a patient with CD8+ PCAECTL, diagnosed as a composite lymphoma with B-cell chronic lymphocytic leukemia, who underwent R-CHOEP (rituximab, cyclophosphamide, doxorubicin, vincristine, and dexamethasone) for six cycles and achieved CR. This patient underwent matched sibling donor HSCT (MSD-HSCT) with an HLA-identical donor (the patient’s brother) and sustained a good response for nine months before ultimately succumbing to CLL relapse and spontaneous bacterial peritonitis [13]. Another 16-year-old patient with CD8+ PCAECTL with cerebral involvement received treatment with high dose MTX, EPOCH (etoposide, prednisolone, vincristine, cyclophosphamide, doxorubicin), and whole-brain radiation, however, continued to experience neurologic symptoms. He underwent cord blood transplantation (HLA mismatched at 3 of 8 loci) with eventual resolution of neurologic symptoms and as of day 136 was doing well with resolution of the previously seen brain lesions on MRI [11].

Of the 24 combined patients in our literature review and the study by Cyrenne et al, 6 received autologous transplants, 1 received cord blood transplantation, and 17 received allogeneic transplantation. One patient received both autologous and allogeneic transplants. Four of the 17 allogeneic transplant cases contained donor information, and of these, two were from HLA-identical sibling donors and two were from matched unrelated donors. MUD-HSCT patients demonstrated durable CRs of 30 and 60 months post-transplant. One HLA-identical alloHSCT patient was in CR 14 months post-transplant, and a second one died of disease after nine months. AutoHSCT patients did not do well, with only two of six patients being alive at the time of publication (one of the patients remained alive with stable disease (SD), while the other later underwent allo-HSCT). Information on response status prior to transplant was available for 14 patients and included 3 CR, 6 partial remissions (PR), 3 SD, and 2 no response. Transplant outcomes did not seem to vary significantly between patients with CR vs PR prior to transplant. However, our data are limited and many of the records in the literature are incomplete. There is also limited data on the durability of CR following transplant. In our review of 24 patients, there are 8 patients with confirmed CR following transplantation, with CR durations reported anywhere from 2 to 60 months. Based on available literature and our own experience, the therapeutic strategy for PCAECTL is first to identify a matched related or unrelated donor. A haploidentical donor can be considered in this setting as well.

## Conclusions

In summary, we described a unique patient withCD8+ PCAECTL, successfully treated with MRD-HCT resulting in a long-term remission of eight years post-transplant. The present case supports the treatment regimen with aggressive induction chemotherapy followed by allogeneic stem cell transplant in younger patients with CD8+ PTAECTL who can tolerate such intensive therapy. One limitation of our review was a lack of data in the current literature on disease status (CR, PR, SD, or progressive disease, PD) before transplantation, a variable which we suspect may have a significant effect on outcomes. Based on our case and the available data, early transplant consult and donor search is one of the key factors in the management of this disease.
